# Prognostic impact of extracranial disease control in HER2+ breast cancer-related brain metastases

**DOI:** 10.1038/s41416-023-02153-w

**Published:** 2023-01-30

**Authors:** Michele Bottosso, Gaia Griguolo, Léa Sinoquet, Maria Cristina Guarascio, Vittoria Aldegheri, Federica Miglietta, Grazia Vernaci, Caterina Barbieri, Fabio Girardi, William Jacot, Valentina Guarneri, Amélie Darlix, Maria Vittoria Dieci

**Affiliations:** 1 Department of Surgery, Oncology and Gastroenterology, University of Padova, Padova, Italy; 2 Division of Oncology 2, Istituto Oncologico Veneto IRCCS, Padova, Italy; 3 Medical Oncology Department, Institut du Cancer de Montpellier, University of Montpellier, Montpellier, France; 4 Radiology Department, Istituto Oncologico Veneto IRCCS, Padova, Italy; 5 Institut de Génomique Fonctionnelle, INSERM, CNRS - University of Montpellier, Montpellier, France

**Keywords:** Breast cancer, Metastasis, CNS cancer, Outcomes research

## Abstract

**Background:**

Brain metastases (BM) are common among HER2+ breast cancer (BC) and prognostic stratification is crucial for optimal management. BC-GPA score and subsequent refinements (modified-GPA, updated-GPA) recapitulate prognostic factors. Since none of these indexes includes extracranial disease control, we evaluated its prognostic value in HER2+ BCBM.

**Methods:**

Patients diagnosed with HER2+ BCBM at Istituto Oncologico Veneto-Padova (2002–2021) and Montpellier Cancer Institute (2001–2015) were included as exploratory and validation cohorts, respectively. Extracranial disease control at BM diagnosis (no disease/stable disease/response vs. progressive disease) was evaluated.

**Results:**

In the exploratory cohort of 113 patients (median OS 12.2 months), extracranial control (*n* = 65, 57.5%) was significantly associated with better OS at univariate (median OS 17.7 vs. 8.7 months, *p* = 0.005) and multivariate analysis after adjustment for BC-GPA (HR 0.61, 95% CI 0.39–0.94), modified-GPA (HR 0.64, 95% CI 0.42–0.98) and updated-GPA (HR 0.63, 95% CI 0.41–0.98). The prognostic impact of extracranial disease control (*n* = 66, 56.4%) was then confirmed in the validation cohort (*n* = 117) at univariate (median OS 20.2 vs. 9.1 months, *p* < 0.001) and multivariate analysis adjusting for BC-GPA (HR 0.41, 95% CI 0.27–0.61), modified-GPA (HR 0.44, 95% CI 0.29–0.67) and updated-GPA (HR 0.42, 95% CI 0.28–0.63).

**Conclusions:**

Extracranial disease control provides independent prognostic information in HER2+ BCBM beyond commonly used prognostic scores.

## Background

Breast cancer (BC) is one of the main causes of brain metastases (BM) among solid tumours. About 30–50% of patients with metastatic BC will eventually develop central nervous system (CNS) involvement [1]. In particular, the incidence of BM in metastatic BC patients has progressively increased over the years [2], mainly due to a better control of systemic disease and longer survival. The risk is especially high in patients with Human Epidermal growth factor Receptor 2 (HER2)-positive or triple negative BC [3, 4].

The diagnosis of BM in BC patients is generally considered as associated with worse prognosis compared to other metastatic sites. However, patients with BCBM represent a heterogeneous population and prognosis is variable according to clinical and histopathologic factors [5–8]. A personalised clinical management with a multimodal integration of local and systemic treatments is therefore recommended [9, 10]. In this complex scenario, an adequate prediction of patient prognosis is needed to support the treatment decision making process. For example, recent guidelines suggest the possibility to delay potentially toxic local therapies such as whole-brain radiotherapy (WBRT) if highly active systemic treatments, such as novel anti-HER2 drugs, are available, with the aim of postponing long-term cognitive side effects in patients for which a long survival is expected [10, 11]. Moreover, anti-HER2 therapies have deeply changed the treatment approach and prognosis of early and advanced HER2-positive BC and the development of anti-HER2 drugs with high intracranial activity has represented one of the major improvements in the treatment of BCBM [12, 13]. As the number of treatment options for patients with HER2-positive BCBM expands and patient prognosis improves, an accurate prognostic prediction is becoming even more relevant, specifically in this subgroup of patients.

Efforts to prognosticate the outcome of patients with BCBM have led to the subsequent development of several different and eventually more precise scores. Some of the first indexes, e.g. the Graded Prognostic Assessment (GPA), were originally developed based on data from cohorts including patients with different primary solid tumours [14]. Subsequently, acknowledging the relevant differences in clinical behaviour and treatment options according to tumour histology, tumour-specific scores have been proposed. In 2012, Sperduto et al. identified, in a cohort of 400 BC patients with BM, Karnofsky Performance Status (KPS), BC subtype (as defined according to hormone receptors and HER2 status) and age as independent prognostic factors and these three variables were included in the breast-specific GPA index [15]. The accuracy of this score was then improved by the addition of number of BM (modified breast-GPA score) as proposed by Subbiah et al. [16] and further updated by the addition of presence/absence of extracranial metastases (updated breast-GPA) [17].

It should however be acknowledged that all these prognostic scores were designed based on data from patients’ cohorts including all BC subtypes (as defined according to hormone receptors and HER2 status). As differences in biology, clinical history and treatment options among BC subtypes increase, further development from breast-specific prognostic scores to BC subtype-specific prognostic scores may be required to account for this evolution. For example, new targeted therapeutic options for patients with HER2-positive BCBM have significantly improved intracranial disease control, thus potentially enhancing the prognostic impact of extracranial disease control (beyond the simple presence or absence of extracranial disease), specifically in this subgroup [18]. Moreover, extracranial disease control also impacts treatment decision in this setting.

This study was designed to assess and validate the potential prognostic impact of extracranial disease control, beyond commonly used prognostic scores, specifically in patients with HER2-positive BCBM.

## Methods

### Patients

Consecutive patients newly diagnosed with BM from HER2-positive BC at Istituto Oncologico Veneto - Padova (Italy) between 2002 and 2021 were included as exploratory cohort. Patients newly diagnosed with HER2-positive BCBM at Montpellier Cancer Institute - Montpellier (France) between 2001 and 2015 were included as validation cohort. Inclusion criteria were: histologically proven HER2-positive BC according to current ASCO/CAP recommendations [19], age >18 years at time of BC diagnosis, intraparenchymal BM radiologically confirmed using contrast-enhanced cerebral computed tomography scan and/or magnetic resonance imaging of the brain, availability of key prognostic factors needed to calculate GPA prognostic scores (age, KPS, tumour subtype, presence of extracranial disease). Patients with diagnosis of leptomeningeal carcinomatosis alone, in the absence of intraparenchymal BM, were excluded, while patients with intraparenchymal BM who were also diagnosed with leptomeningeal disease at time or after BM diagnosis were included.

Demographic, clinicopathologic and treatment data were retrospectively collected from medical charts in a dedicated database. Oestrogen receptor (ER) and progesterone receptor (PgR) expression were determined by immunohistochemistry; positivity was defined as immunohistochemistry staining in at least 1% of tumour cells. The tumour was considered HER2 positive if scored 3+ by immunohistochemistry or if the HER2 gene was amplified by fluorescence or chromogenic in situ hybridisation (FISH/CISH) for immunohistochemistry 2+ cases.

For each patient, breast-GPA, modified breast-GPA and updated breast-GPA were calculated based on published criteria [15–17]. Each score divides patients into four groups: 0–1.0, 1.5–2.0, 2.5–3.0, and 3.5–4.0; a score of 4.0 is associated with best prognosis for all three scores. Radiological extracranial disease control at the time of BM diagnosis (disease control: no evidence/stable disease/partial response/complete response of extracranial disease vs. progression of extracranial disease) was evaluated according to RECIST 1.1 criteria.

This study was reviewed and approved by the involved Institutional Review Boards and Ethics Committees. Where necessary according to local regulation, written informed consent was obtained from participants. The study was conducted in accordance with the Declaration of Helsinki.

### Statistical analysis

Statistical analysis was performed using IBM SPSS (version 25). Descriptive statistics were performed for patients’ demographics and clinical characteristics. The Chi-squared test (*χ*^2^) test was used to study association between variables.

Overall survival from BM diagnosis (OS) was defined as time from BM diagnosis to death from any cause. Patients alive without event at cut-off date of this analysis were censored at date of last follow-up. OS was estimated using the Kaplan–Meier method and reported with its 95% confidence intervals (95% CIs). The log-rank test was used to compare OS between groups. Univariate and multivariate Cox regression modelling for proportional hazards was used to calculate hazard ratios and their 95% CI. Likelihood ratio test *χ*^2^ and *p* values were generated comparing consolidate prognostic scores with and without the addition of extracranial disease control in order to test for the additional prognostic value of extracranial disease control.

All reported *p* values were two-sided and significance level was set at 5% (*p* < 0.05).

## Results

### Patient characteristics in the exploratory cohort

Overall, 113 HER2-positive BC patients diagnosed with BM were identified and included in the exploratory cohort. Main patient and tumour characteristics are summarised in Table 1.

**Table 1:** Patient and tumour characteristics at time of brain metastasis diagnosis in the exploratory and validation cohort.

	Exploratory cohort *N* = 113 (%)	Validation cohort *N* = 117 (%)
Median age at BC diagnosis (range)	51 (23–80)	50 (22–79)
Median age at BM diagnosis (range)	55 (26–84)	54 (31–81)
Age at BM diagnosis		
≤50 years	35 (31.0)	44 (37.6)
>50 years	78 (69.0)	73 (62.4)
Tumour histology		
Ductal	96 (84.9)	100 (85.5)
Lobular	9 (8.0)	7 (6.0)
Other	7 (6.2)	10 (8.5)
Missing	1 (0.9)	0 (0)
Histologic grade		
G1–G2	25 (22.1)	48 (41.0)
G3	83 (73.5)	55 (47.0)
Missing	5 (4.4)	14 (12.0)
HR status		
Positive	66 (58.4)	51 (43.6)
Negative	47 (41.6)	66 (56.4)
Stage at first diagnosis of BC		
I	30 (26.5)	13 (11.1)
II	28 (24.8)	23 (19.7)
III	36 (31.9)	33 (28.2)
IV	1 (0.9)	38 (32.5)
Missing	18 (15.9)	10 (8.5)
Karnofsky performance status		
90–100	27 (23.9)	27 (23.1)
70–80	58 (51.3)	61 (52.1)
60	13 (11.5)	17 (14.5)
≤50	15 (13.3)	12 (10.3)
BM at first relapse		
Yes	27 (23.9)	28 (23.9)
No	86 (76.1)	89 (76.1)
Number of BM		
1	37 (32.7)	26 (22.2)
2	13 (11.5)	19 (16.3)
3	13 (11.5)	6 (5.1)
≥4	50 (44.3)	66 (56.4)
Extracranial metastases		
Absent	16 (14.2)	105 (89.7)
Present	97 (85.8)	12 (10.3)
Extra-CNS disease control		
Yes	65 (57.5)	66 (56.4)
No	48 (42.5)	51 (43.6)
Extra-CNS disease status at BM		
NED	16 (14.1)	12 (10.3)
CR	1 (0.9)	0 (0)
PR	8 (7.1)	5 (4.3)
SD	40 (35.4)	49 (41.9)
PD	48 (42.5)	51 (43.5)
Breast-GPA score		
0.0–1.0	0 (0)	0 (0)
1.5–2.0	14 (12.4)	17 (14.5)
2.5–3.0	55 (48.7)	58 (49.6)
3.5–4.0	44 (38.9)	42 (35.9)
Modified breast-GPA score		
0.0–1.0	3 (2.7)	6 (5.1)
1.5–2.0	30 (26.5)	24 (20.5)
2.5–3.0	57 (50.4)	70 (59.8)
3.5–4.0	23 (20.4)	17 (14.6)
Updated breast-GPA score		
0.0–1.0	0 (0)	0 (0)
1.5–2.0	40 (35.4)	42 (35.9)
2.5–3.0	57 (50.4)	66 (56.4)
3.5–4.0	16 (14.2)	9 (7.7)

*BC* breast cancer, *BM* brain metastases, *HR* hormone receptor, *CNS* central nervous system, *NED* not evidence of disease, *CR* complete response, *PR* partial response, *SD* stable disease, *PD* progressive disease, *GPA* graded prognostic assessment.

Median age at the time of BM diagnosis was 55 years (range 26–84). More than half of the tumours (*n* = 66, 58.4%) were hormone receptor positive. Most patients (*n* = 85, 75.2%) had a conserved performance status (KPS ≥ 70) at the time of BM diagnosis. Approximately one third of patients presented a single BM while 44% had more than 3 brain lesions at time of BM diagnosis. Moreover, although the large majority of patients presented extra-CNS lesions (*n* = 97, 85.8%), in most cases extracranial disease was under control (*n* = 65, 57.5%) at the time of BM diagnosis. Median time between BM diagnosis and extracranial status assessment was 6.0 days (95% CI 0.0–12.2). Among patients with systemic disease control at BM diagnosis, 30.8% (*n* = 20) eventually developed an extracranial progression subsequently, with a median time to extracranial progression of 5.2 months (96% CI 3.2–7.2) in this subgroup of patients. Concomitant leptomeningeal metastasis at BM diagnosis were present in 8 (7.1%) patients (3 with extracranial disease control and 5 with extracranial progression).

After BM diagnosis, the majority of patients received active therapy for BCBM with at least one treatment modality, local or systemic, while only 13 patients (11.5%) were treated with best supportive care alone. Most patients (*n* = 91, 80.5%) received at least one line of systemic treatment, which included an anti-HER2 targeted agent in most cases (*n* = 79, 69.9%). Comprehensive data regarding treatment received by patients included in the exploratory cohort are reported in Supplementary Table 1.

### Prognostic factors for overall survival in the exploratory cohort: univariate and multivariate analysis

With a median follow-up of 53.3 months (95% CI 33.9–72.6), 92 patients (81.4%) had died. Median OS from the time of BM diagnosis was 12.2 months (95% CI 6.2–18.1). Among the 64 patients for whom the cause of death was available, 73.4% (*n* = 47) died because of intracranial progression (60.7% among patients with extracranial progression at BM diagnosis and 83.3% among patients with extracranial control; *p* = 0.042).

The association between several known prognostic factors and OS from BM diagnosis was investigated using univariate Cox regression modelling (Table 2). As expected, Karnofsky Performance Status, number of BM and presence of extracranial metastases were all significantly associated with OS from BM diagnosis. All the prognostic score tested (breast-GPA, modified breast-GPA, updated breast-GPA) were significantly associated with OS from BM diagnosis.

**Table 2:** Univariate Cox proportional hazards model for OS in the exploratory cohort.

	Median OS, months (95% CI)	Univariate Cox hazard ratio (95% CI)	*p* value
Age			
≤50 years	18.8 (11.6–26.6)	0.78 (0.49–1.23)	0.283
>50 years	9.8 (6.3–13.2)	Ref.	
HR status			
Positive	16.1 (8.1–24.1)	0.67 (0.43–1.02)	0.061
Negative	8.8 (3.7–13.9)	Ref.	
Karnofsky performance status			
90–100	29.0 (20.4–37.6)	0.08 (0.04–0.17)	**<0.001**
80	13.7 (4.3–23.2)	0.12 (0.06–0.25)	
70	8.7 (3.0–14.3)	0.18 (0.08–0.38)	
60	4.2 (0.0–13.7)	0.31 (0.13–0.71)	
≤50	1.7 (0.9–2.5)	Ref.	
Stage at BC diagnosis			
I–III	11.4 (5.0–17.8)	1.16 (0.71–1.90)	0.545
IV	17.7 (0.0–35.4)	Ref.	
Leptomeningeal metastases at BM diagnosis			
No	13.7 (6.4–21–1)	0.59 (0.342–1.02)	0.056
Yes	3.5 (2.56–4.47)	Ref.	
BM at first relapse			
Yes	19.5 (9.6–29.4)	0.73 (0.44–1.20)	0.214
No	9.2 (2.3–16.0)	Ref.	
Number of BM			
1	6.0 (16.8–40.3)	0.33 (0.19–0.56)	**<0.001**
2	5.4 (0.0–17.9)	0.77 (0.40–1.49)	
3	4.5 (0.0–13.2)	0.51 (0.63–2.52)	
≥4	2.4 (2.3–11.6)	Ref.	
Extracranial metastases			
Absent	28.6 (3.7–53.4)	0.45 (0.23–0.88)	**0.017**
Present	10.4 (3.4–17.4)	Ref.	
Extra-CNS disease control			
Yes	17.7 (9.0–26.5)	0.55 (0.36–0.54)	**0.005**
No	8.7 (1.8–15.6)	Ref.	
Breast-GPA score			
1.5–2.0	2.0 (0.0–4.5)	5.51 (2.83–10.75)	**<0.001**
2.5–3.0	8.7 (2.8–14.5)	2.45 (1.52–3.95)	
3.5–4.0	23.7 (12.2–35.2)	Ref.	
Modified breast-GPA score			
0.0–1.0	1.4 (0.0–2.9)	18.78 (4.96–71.06)	**<0.001**
1.5–2.0	3.0 (1.4–4.7)	6.67 (3.20–13.91)	
2.5–3.0	12.2 (7.8–16.6)	3.54 (1.80–6.97)	
3.5–4.0	31.0 (15.0–47.1)	Ref.	
Updated breast-GPA score			
1.5–2.0	3.2 (0.6–5.8)	6.15 (2.95–12.84)	**<0.001**
2.5–3.0	18.0 (9.6–26.4)	2.21 (1.10–4.44)	
3.5–4.0	29.0 (26.1–31.9)	Ref.	

*OS* overall survival, *HR* hormone receptor, *BM* brain metastases, *CNS* central nervous system, *GPA* graded prognostic assessment.

Bold values represent statistically significant associations (*p* < 0.05).

**Fig. 1:**
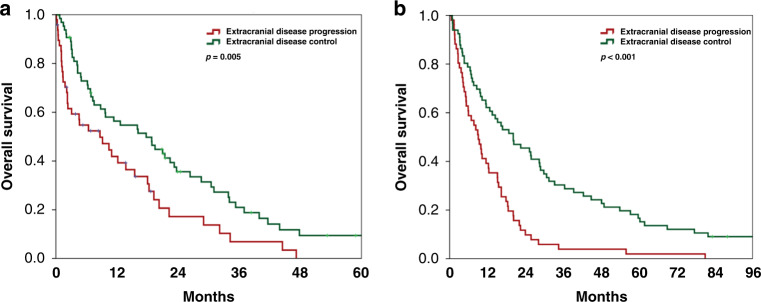
Overall survival according to extracranial disease control. Kaplan–Meier curves showing the impact of extracranial disease control on overall survival from time of brain metastases diagnosis in the exploratory cohort (**a**) and in the validation cohort (**b**).

In addition, extra-CNS disease control was also identified as a significant prognostic factor at univariate analysis with a median OS of 17.7 and 8.7 months for patients with or without extracranial disease control, respectively (log-rang *p* = 0.005; Fig. 1a).

To evaluate whether extra-CNS disease control added independent prognostic information to each of the three prognostic scores (breast-GPA, modified breast-GPA, updated breast-GPA), a multivariate analysis was performed. Each prognostic score was tested separately to account for the significant overlap among the different prognostic scores.

Extra-CNS disease control maintained its independent prognostic value after correction for each of the validated prognostic scores: breast-GPA score (HR 0.61, 95% CI 0.39–0.94, *p* = 0.025), modified breast-GPA score (HR 0.64, 95% CI 0.42–0.98, *p* = 0.039) and updated breast-GPA score (HR 0.63, 95% CI 0.41–0.98, *p* = 0.040). The likelihood ratio test was also used to evaluate for the added prognostic value of extracranial disease control to each GPA score, showing an improvement in terms of prognosis prediction by the inclusion of extracranial disease control (Table 3 and Supplementary Table 4).

**Table 3:** Prognostic impact of extracranial disease control after correction for each of the three consolidated prognostic scores (multivariate analysis) in the exploratory and in the validation cohorts.

		Corrected for breast-GPA				Corrected for modified-GPA				Corrected for updated-GPA			
		HR (95% CI)	*p* value	LR *χ*^2^	LR *p* value	HR (95% CI)	*p* value	LR *χ*^2^	LR *p* value	HR (95% CI)	*p* value	LR *χ*^2^	LR *p* value
Exploratory cohort	Extra-CNS control												
	Yes	0.61 (0.39–0.94)	**0.025**	4.89	**0.027**	0.64 (0.42–0.98)	**0.039**	4.15	**0.042**	0.63 (0.41–0.98)	**0.040**	4.13	**0.042**
	No	Ref.				Ref.				Ref.			
Validation cohort	Extra-CNS control												
	Yes	0.41 (0.27–0.61)	**<0.001**	18.59	**<0.001**	0.44 (0.29–0.67)	**<0.001**	14.02	**<0.001**	0.42 (0.28–0.63)	**<0.001**	17.60	**<0.001**
	No	Ref.				Ref.				Ref.			

*GPA* graded prognostic assessment, *HR* hazard ratio, *CNS* central nervous system, *LR* likelihood ratio.

Bold values represent statistically significant associations (*p* < 0.05).

### Validation cohort: patient characteristics and prognostic factors for overall survival

To validate our results, 117 patients diagnosed with HER2-positive BCBM at the Montpellier Cancer Institute between 2001 and 2015 were included as validation cohort (Table 1). Similarly to the exploratory cohort, median age at BC diagnosis was 50 years (range 22–79). Almost half of the tumours (*n* = 51, 43.6%) were hormone receptor positive and most patients (*n* = 88, 75.2%) had a conserved performance status (KPS ≥ 70) at time of BM diagnosis. More than half of cases (*n* = 66, 56.4%) presented more than 3 brain lesions at time of BM diagnosis. Although the large majority of patients presented extra-CNS lesions (*n* = 105, 89.7%), in most cases extracranial disease was under control (*n* = 66, 56.4%) at time of BM diagnosis and median time between BM diagnosis and extracranial disease assessment was 6.0 day (95% CI 1.8–10.2). Among patients with systemic disease control at BM diagnosis, 45.4% (*n* = 30) eventually developed an extracranial progression and median time to extracranial progression was 5.0 months (96% CI 3.9–6.1). Six (5.1%) patients presented concomitant leptomeningeal metastases (4 among patients with extracranial disease control and 2 among patient with extracranial progression). Most patients (*n* = 102, 87.9%) in the validation cohort received systemic treatments (which included an anti-HER2 therapy in 83.6% of cases) and radiotherapy (*n* = 101, 86.3%). Detailed data regarding treatment received by patients included in the validation cohort are presented in Supplementary Table 2.

The validation cohort presented a longer median follow-up of 96.6 months (95% CI 84.4–108.8), at which time 111 patients (94.9%) had died. Median OS in the validation cohort was 12.7 months (95% CI 8.5–17.7), very similar to what observed in the exploratory cohort. Intracranial progression represented the cause of death for 52 of the 91 (57.1%) patients for whom this information was available (43.6% among patients with extracranial progression at BM diagnosis and 67.3% among patients with extracranial control; *p* = 0.023).

The association between known prognostic factors and OS from BM diagnosis in the validation cohort was investigated using univariate Cox regression modelling (Supplementary Table 3). Karnofsky performance status and all GPA scores were associated with OS, while number of BM and presence of extracranial metastases were not confirmed as significant predictor of OS in the validation cohort. In addition, the prognostic role of extracranial disease control was confirmed with a median OS of 20.2 months and 9.1 months in patients with and without extracranial disease control, respectively (log-rank *p* < 0.001; Fig. 1b).

**Fig. 2:**
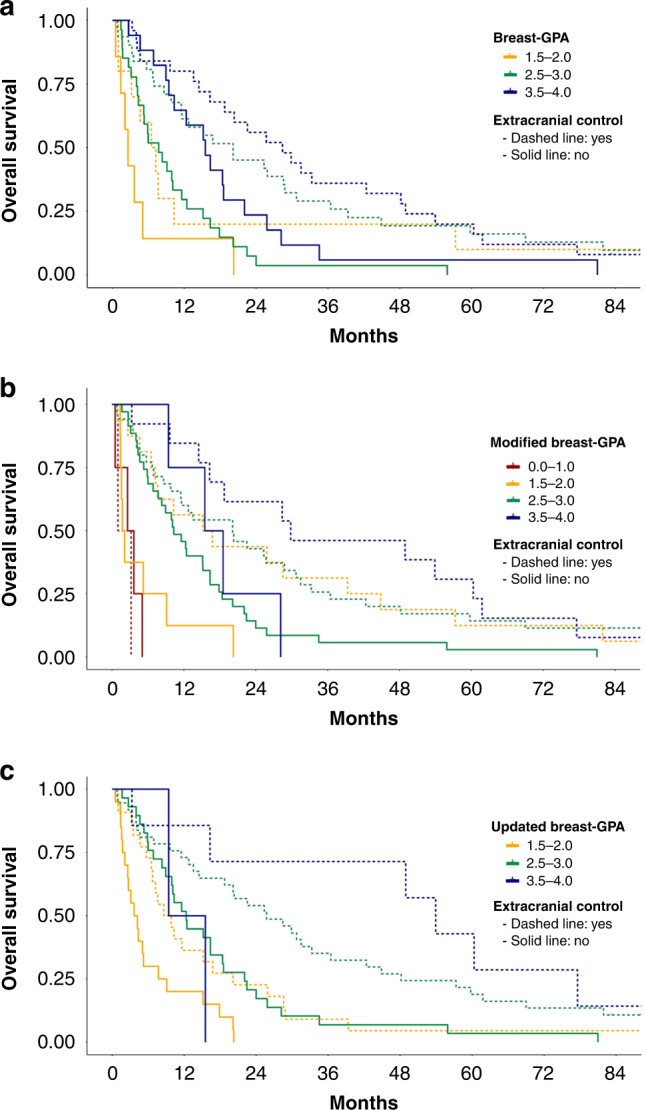
Overall survival in the validation cohort according to extracranial disease control and GPA scores. Overall survival from time of brain metastases diagnosis in the validation cohort according to extracranial disease status (extracranial control in dashed lines, extracranial progression in solid lines) and breast-GPA (**a**), modified breast-GPA (**b**) and updated breast-GPA (**c**) prognostic groups.

Therefore, we tested in multivariate analysis if extra-CNS disease control added independent prognostic information to each of the three prognostic scores (breast-GPA, modified breast-GPA, updated breast-GPA). In the validation cohort, the prognostic impact of extracranial disease control was maintained at multivariate analysis after adjusting for breast-GPA (HR 0.41, 95% CI 0.27–0.61, *p* < 0.001), modified breast-GPA (HR 0.44, 95% CI 0.29–0.67, *p* < 0.001) and updated breast-GPA (HR 0.42, 95% CI 0.28–0.63, *p* < 0.001). The added prognostic value of extracranial disease control was also confirmed applying the likelihood ratio test to the model adding extracranial disease control to each GPA score (Table 3 and Supplementary Table 4). A graphic representation of OS in the validation cohort according to extracranial disease control in each prognostic group of the three GPA scores is shown in Fig. 2.

## Discussion

The therapeutic scenario of HER2-positive BC patients diagnosed with BM has radically changed over the last few decades with the introduction of several novel HER2-targeted agents with relevant intracranial activity alongside with improvements in locoregional treatment modalities [18]. In the context of increased chances of intracranial disease control, the prognostic impact of extracranial disease control (beyond the simple presence or absence of extracranial disease) might potentially be enhanced. Therefore, we designed the present study to evaluate the potential prognostic impact of extracranial disease control, beyond commonly used prognostic scores, specifically in patients with HER2-positive BCBM. To that end, we assessed the prognostic impact of extracranial disease control in two separate large cohorts of HER2+ BCBM patients and successfully identified in both cohorts that it adds independent prognostic information to commonly used prognostic indexes.

It’s worth noticing that in both cohorts the majority of patients did not present a progressive extracranial disease at the time of BM diagnosis. This information carries important therapeutic implications: on one hand, current guidelines recommend not to switch systemic treatments in case of isolated brain progression; on the other hand, more aggressive locoregional therapies such as neurosurgery are generally discouraged in case of concomitant progressive systemic disease [9, 11]. Extracranial disease control should therefore be always assessed at the moment of BM diagnosis and should be taken into account in the multidisciplinary management of these patients.

The prognostic impact of extracranial disease control has been previously assessed in cohorts including patients with BM from different solid tumours; however, contradictory results have been reported [20–22] and extracranial disease control has not been univocally established as an independent prognostic factor among patients with BM. Moreover, even if the recently updated breast-GPA [17] has been modified to include the presence or absence of extra-CNS lesions, the potential prognostic impact of extracranial disease control or progression was not evaluated.

Previous studies already suggested the prognostic role of extracranial disease control in patients with HER2-positive BCBM. In 2009, Park et al. reported extracranial disease control as a significant prognostic factor at multivariate analysis in 77 patients diagnosed with HER2+ BCBM after the introduction of anti-HER2 targeted therapies. However, this study did not address the added value of extracranial disease control as compared to validated prognostic scores and the findings were not validated in an independent cohort [23]. More recently, Noteware et al. reported data regarding the potential impact of extracranial disease status in a cohort of 153 patients with HER2-positive BCBM treated with CNS radiation. In this cohort, OS from first BM diagnosis was significantly worse for patients with progressive extracranial disease as compared to patients with stable/responding extracranial disease or no extracranial disease (log-rank *p* = 0.008) [24]. Still, additional value of extracranial disease control as compared to prognostic scores was not reported, neither validated in an independent cohort.

These findings highlight the importance of an accurate selection of homogeneous groups of patients with BM in order to better capture different clinical behaviours and treatment availabilities and therefore to identify more specific prognostic factors. In this context, even BC-specific scores may not be sufficiently precise to adequately describe prognosis in all the different BC subgroups. Indeed, according to the breast-GPA and the updated-GPA, no patients with HER2-positive BCBM can be classified in the worst prognosis category. Nevertheless, a wide range of outcomes has been described also among patients with HER2-positive BCBM [15, 17]. The accurate prognostic assessment of BC patients diagnosed with BM is not trivial: in this challenging clinical scenario, where different treatment modalities are usually integrated, prognostic scores can aid physicians in treatment choice. In particular, as HER2 positivity is considered a positive prognostic factor for BC patients with BM and, in some cases with expected good prognosis, delay of local treatment is now being proposed by guidelines, an adequate prediction of outcome for these patients is even more crucial for treatment planification [3].

Our findings demonstrate in two independent patient cohorts that extracranial disease control carries significant independent prognostic information, beyond commonly used prognostic score. This observation supports the integration of this feature in future prognostic scores specifically designed for HER2-positive BC patients with BM. Indeed, the impact of extracranial control on patients’ outcome could become even more relevant in the next few years as the number of anti-HER2 targeted agents continues to increase. Recently approved drugs, such as trastuzumab deruxtecan and tucatinib, demonstrated a remarkable intracranial disease response rate [25–27] while several new strategies are being tested in dedicated clinical trials (NCT04539938, NCT04512261, NCT04639271). Moreover, the introduction in clinical practise of new drugs able to change the history of HER2-positive disease highlights the need of continuously reassessing prognostic factors in contemporary cohorts.

## Supplementary information

Supplementary material

## Data Availability

Data are available upon reasonable request to the corresponding author.
