# Supplementary material for: Prognostic impact of extracranial disease control in HER2+ breast cancer-related brain metastases
Source: Br J Cancer. 2023 Jan 30;128(7):1286–93. doi: 10.1038/s41416-023-02153-w (PMC10049979; doi:10.1038/s41416-023-02153-w)
Supplement: Supplementary file 1 — Supplementary material [file 41416_2023_2153_MOESM1_ESM.docx]

Supplementary Table 1. Treatment received after BM diagnosis in the exploratory cohort.

|  | | N. of patients (%) |
| --- | --- | --- |
| Neurosurgical resection | |  |
|  | Yes | 21 (18.6) |
|  | No | 92 (81.4) |
| Radiotherapy after BM diagnosis | |  |
|  | Yes | 80 (70.8) |
|  | No | 33 (29.2) |
| SRS | |  |
|  | Yes | 23 (20.4) |
|  | No | 90 (79.6) |
| WBRT | |  |
|  | Yes | 59 (52.2) |
|  | No | 54 (47.8) |
| Ongoing line of therapy at BM diagnosis | |  |
|  | None | 28 (24.8) |
|  | 1^st^ | 21 (18.6) |
|  | 2^nd^ | 28 (24.8) |
|  | 3^rd^ | 17 (15.0) |
|  | >3^rd^ | 19 (16.8) |
| Systemic treatment after BM diagnosis | |  |
|  | Yes | 91 (80.5) |
|  | No | 22 (19.5) |
| Switch from previous systemic treatment at BM diagnosis* | |  |
|  | Yes | 16 (24.6) |
|  | No | 45 (69.2) |
|  | Missing | 4 (6.2) |
| Chemotherapy after BM diagnosis | |  |
|  | Yes | 70 (61.9) |
|  | No | 43 (38.1) |
| Anti-HER2 therapy after BM diagnosis | |  |
|  | Yes | 79 (69.9) |
|  | No | 34 (30.1) |
| Hormone therapy after BM diagnosis | |  |
|  | Yes | 22 (19.5) |
|  | No | 91 (80.5) |

*among patients with extracranial disease control
Abbreviations: BM brain metastases, SRS stereotactic radiosurgery, WBRT whole brain radiotherapy, HER2 human epidermal growth factor receptor 2

Supplementary Table 2. Treatment received after BM diagnosis in the validation cohort.

|  | | N. of patients (%) |
| --- | --- | --- |
| Neurosurgery | |  |
|  | Yes | 18 (15.4) |
|  | No | 99 (84.6) |
| Radiotherapy after BM diagnosis | |  |
|  | Yes | 101 (86.3) |
|  | No | 16 (13.7) |
| SRS | |  |
|  | Yes | 25 (21.6) |
|  | No | 91 (78.4) |
| WBRT | |  |
|  | Yes | 90 (77.6) |
|  | No | 26 (22.4) |
| Ongoing line of therapy at BM diagnosis | |  |
|  | None | 31 (26.5) |
|  | 1^st^ | 50 (42.7) |
|  | 2^nd^ | 14 (12.0) |
|  | 3^rd^ | 11 (9.4) |
|  | >3^rd^ | 11 (9.4) |
| Systemic treatment after BM diagnosis | |  |
|  | Yes | 102 (87.9) |
|  | No | 14 (12.1) |
| Switch from previous systemic treatment at BM diagnosis* | |  |
|  | Yes | 35 (53.0) |
|  | No | 31 (47.0) |
| Chemotherapy after BM diagnosis | |  |
|  | Yes | 93 (80.2) |
|  | No | 23 (19.8) |
| Anti-HER2 therapy after BM diagnosis | |  |
|  | Yes | 97 (83.6) |
|  | No | 19 (16.4) |
| Hormone therapy after BM diagnosis | |  |
|  | Yes | 14 (12.1) |
|  | No | 102 (87.9) |

*among patients with extracranial disease control
Abbreviations: BM brain metastases, SRS stereotactic radiosurgery, WBRT whole brain radiotherapy, HER2 human epidermal growth factor receptor 2Supplementary Table 3. Univariate Cox Proportional Hazards Model for OS in the validation cohort

|  | | | Median OS, months  (95% CI) | Univariate Cox Hazard ratio  (95% CI) | p-value |
| --- | --- | --- | --- | --- | --- |
| Age | | |  |  |  |
|  | | ≤50 years | 18.4 (10.5-26.3) | 0.70 (0.47-1.03) | 0.068 |
|  | | >50 years | 9.1 (5.9-12.3) | Ref |  |
| HR status | | |  |  | 0.462 |
|  | Positive | | 14.5 (9.7-19.2) | 0.87 (0.59-1.27) |  |
|  | Negative | | 11.5 (5.4-17.5) | Ref |  |
| Karnofsky Performance Status | | |  |  | **<0.001** |
|  | 90-100 | | 18.4 (11.5-25.3) | 0.24 (0.12-0.48) |  |
|  | 70-80 | | 15.2 (8.5-21.9) | 0.30 (0.16-0.57) |  |
|  | 60 | | 9.1 (5.0-13.2) | 0.35 (0.16-0.77) |  |
|  | ≤50 | | 2.6 (0.8-4.4) | Ref |  |
| Stage at BC diagnosis | | |  |  | 0.221 |
|  | I-III | | 12.3 (7.9-16.7) | 1.29 (0.86-1.93) |  |
|  | IV | | 16.4 (5.5-27.2) | Ref. |  |
| Leptomeningeal metastases at BM diagnosis | | |  |  | 0.270 |
|  | Yes | | 16.4 (13.6-19.1) | 0.61 (0.25-1.49) |  |
|  | No | | 12.3 (8.4-16.2) | Ref. |  |
| BM at first relapse | | |  |  | 0.747 |
|  | No | | 14.5 (9.6-19.3) | 0.93 (0.60-1.43) |  |
|  | Yes | | 9.6 (2.6-16.7) | Ref |  |
| Number of BM | | |  |  | 0.070 |
|  | 1 | | 16.4 (10.5-22.2) | 0.55 (0.34-0.90) |  |
|  | 2 | | 9.6 (6.5-12.8) | 1.00 (0.60-1.68) |  |
|  | 3 | | 18.8 (5.7-31.8) | 0.57 (0.23-1.42) |  |
|  | ≥4 | | 11.5 (7.1-15.9) | Ref |  |
| Extracranial metastases | | |  |  | 0.467 |
|  | Absent | | 10.3 (0.0-36.9) | 0.80 (0.44-1.46) |  |
|  | Present | | 12.7 (8.3-17.2) | Ref |  |
| Extra-CNS disease control | | |  |  | **<0.001** |
|  | Yes | | 20.2 (10.1-30.3) | 0.44 (0.29-0.65) |  |
|  | No | | 9.1 (5.9-12.3) | Ref |  |
| Breast-GPA score | | |  |  | **0.022** |
|  | 1.5-2.0 | | 4.6 (2.1-7.1) | 2.23 (1.24-4.00) |  |
|  | 2.5-3.0 | | 11.5 (8.2-14.8) | 1.36 (0.90-2.05) |  |
|  | 3.5-4.0 | | 18.8 (12.2-25.3) | Ref |  |
| Modified Breast-GPA score | | |  |  | **<0.001** |
|  | 0.0-1.0 | | 2.6 (0.0-5.2) | 13.97 (4.99-39.12) |  |
|  | 1.5-2.0 | | 7.6 (3.1-12.2) | 1.72 (0.90-3.25) |  |
|  | 2.5-3.0 | | 12.5 (8.7-16.2) | 1.53 (0.88-2.65) |  |
|  | 3.5-4.0 | | 28.2 (14.9-41.4) | Ref |  |
| Updated Breast-GPA score | | |  |  | **<0.001** |
|  | 1.5-2.0 | | 6.5 (3.8-9.2) | 3.40 (1.57-7.38) |  |
|  | 2.5-3.0 | | 18.4 (12.5-24.3) | 1.53 (0.73-3.22) |  |
|  | 3.5-4.0 | | 49.0 (0.0-144.4) | Ref |  |

Abbreviations: OS overall survival, HR hormone receptor, BM brain metastases, CNS central nervous system, GPA graded prognostic assessment

Supplementary Table 4. Multivariate analysis of prognostic factors in the exploratory and in the validation cohorts

|  |  |  | HR  (95% CI) | p-value |
| --- | --- | --- | --- | --- |
| Exploratory cohort* | Number of BM | 1  2  3  ≥4 | 0.46 (0.25-0.83)  0.99 (0.49-1.99)  1.91 (0.91-4.0)  Ref. | 0.004 |
|  | KPS | 90-100  70-80  60  ≤50 | 0.09 (0.04-0.20)  0.16 (0.08-0.32)  0.20 (0.08-0.50)  Ref. | <0.001 |
|  | Extracranial disease control | Present  Absent | 0.58 (0.36-0.92)  Ref. | 0.021 |
| Validation cohort | KPS | 90-100  70-80  60  ≤50 | 0.22 (0.11-0.44)  0.25 (0.13-0.49)  0.31 (0.14-0.68)  Ref. | <0.001 |
|  | Extracranial disease control | Present  Absent | 0.41 (0.28-0.62)  Ref. | <0.001 |

*The variable presence/absence of extracranial metastases was excluded from multivariate analysis because associated with the variable extracranial disease control
Abbreviations: BM brain metastases, KPS Karnofsky Performance Status, HR hazard ratio, CI confidence interval
